# MicroRNA-451a, microRNA-34a-5p, and microRNA-221-3p as predictors of response to antidepressant treatment

**DOI:** 10.1590/1414-431X20187212

**Published:** 2018-05-17

**Authors:** Wei-Hong Kuang, Zai-Quan Dong, Lian-Tian Tian, Jin Li

**Affiliations:** 1 Department of Psychiatry and Mental Health Center, West China Hospital, Sichuan University, Chengdu, China; 2 Research Centre for Public Health and Preventive Medicine, West China School of Public Health, No. 4 West China Teaching Hospital, Sichuan University, Chengdu, China

**Keywords:** MicroRNA-451a, MicroRNA-34a-5p, MicroRNA-221-3p, Depression, Hamilton Depression Scale, Paroxetine

## Abstract

Aberrant expression of microRNAs (miRNAs) has been shown to be involved in early observations of depression. The aim of this study was to determine if serum levels of miRNA-451a, miRNA-34a-5p, and miRNA-221-3p can serve as indicators of disease progression or therapeutic efficacy in depression. We collected data from 84 depressed patients and 78 control volunteers recruited from the medical staff at the West China Hospital. Depression severity was rated using the 24-item Hamilton Depression Scale (HAMD). Serum miRNA-451a, miRNA-34a-5p, and miRNA-221-3p levels were determined in samples from the depressed patients before and 8 weeks after antidepressant treatment as well as in samples from controls. Compared with the controls, the patients had lower miRNA-451a levels, higher miRNA-34a-5p and miRNA-221-3p levels, and increased HAMD scores whether they underwent antidepressant treatment or not. Eight weeks after antidepressant treatment, the patients exhibited increased miRNA-451a levels, decreased miRNA-34a-5p and miRNA-221-3p levels, and reduced HAMD scores. The serum level of miRNA-451a was negatively correlated with HAMD scores of the patients, while the serum levels of miRNA-34a-5p and miRNA-221-3p were positively correlated with HAMD scores whether the patients underwent antidepressant treatment or not. Paroxetine was markedly effective in 50 patients who also displayed an increased level of miRNA-451a but reduced levels of miRNA-34a-5p and miRNA-221-3p. In contrast, paroxetine was moderately effective or ineffective in 34 patients. In conclusion, depressed patients had lower serum miRNA-451a but higher serum miRNA-34a-5p and miRNA-221-3p, and these miRNAs are potential predictors of the efficacy of antidepressants.

## Introduction

Depression is a serious mental disorder characterized by significant and persistent low mood, retardation, aversion to activity, and repeated suicidal thoughts (1). The incidence and recurrence rates of depression are high (2). According to the World Health Organization, depression affects approximately 154 million people worldwide annually (3). By 2020, depression will become the second leading cause of death and disability (4). The manifestation and pathogenesis of depression is very complex, involving genetic, personality, and social factors (5). The risk factors for depression include changes in expression levels of neurotransmitters, susceptibility of gene polymorphisms, and damaged nerve formation function (6). The treatments for depression primarily include antidepressant drugs and psychological treatment (7). Recent evidence suggests that microRNA (miRNA) may be directly or indirectly involved in the onset and development of depression as well as in the treatment of depression (8).

miRNAs are a class of small RNA molecules that are important in the post-transcriptional regulation of gene expression (9) and can regulate central nervous system functions, including cognitive performance, reward feedback, and circadian rhythm (10). Specific miRNA imbalances may cause a range of neurological disorders, such as Alzheimer's disease and schizophrenia (11). A recent study has shown that abnormal heart and brain tissue could release miRNAs into the circulating blood and cerebrospinal fluid, as evidenced by the presence of significantly abnormal expression of miRNAs in the brain tissue of patients with severe depression who committed suicide (12). miRNA-451a is located in chromosome 17q11.2, and can inhibit the proliferation and growth of cells (13). Research has shown that in cancer tissues, the expression of miRNA-451a is down-regulated (14). In addition, miRNA-451 was reported to affect the pathogenesis of autism spectrum disorders, and promote neuronal injury in genetically predisposed individuals (15). Studies have shown that serum miRNA-221-3p (16) and miRNA-34a (17) were significantly decreased in depression patients after taking antidepressants.

Therefore, the aim of this study was to compare the miRNA-451a, miRNA-34a-5p, and miRNA-221-3p content in individuals with and without depression, and in patients with depression before and after antidepressant treatment, providing evidence for the early diagnosis and treatment efficacy of depression.

## Material and Methods

### Ethics approval

The study protocol was approved by the Hospital Institutional Review Board of the West China Hospital, Sichuan University. All participants in the study provided written informed consent.

### Study participants

We collected data from 84 patients diagnosed with depression from January 2014 to June 2015 at the West China Hospital, Sichuan University. The inclusion criteria were as follows: 1) meeting diagnostic criteria for depression according to the US Diagnostic and Statistical Manual of Mental Disorders 4th Edition (DSM-IV) (18); 2) no antidepressant therapy for new or previous diagnosis of depression within two weeks prior to enrollment; 3) rated >20 on the 24-item Hamilton Depression Scale (HAMD) (19); and 4) consent of the patient or family members. The exclusion criteria were as follows: 1) serious physical illness or a history of alcohol or drug abuse; 2) a history of serious heart disease and severe liver or kidney dysfunction; 3) pregnant or lactating women; and 4) a history of manic episodes. Seventy-eight healthy controls were recruited from the same community and during the same time. The controls had HAMD-24 scores of less than 8 points and they had no history of severe traumatic brain disorders or other mental disorders, and no history of suicide attempt. Pregnant or lactating women were not included.

### HAMD rating of depression severity

HAMD is the most widely used scale to assess clinical depression (20), mainly for the depressive symptoms in adult patients. Our study used the 24-item version, while most other studies used a 5-item scale, with 0 to 4 representing “none”, “mild”, “moderate”, “severe”, and “very heavy” depression symptom. A few studies have used a 3-item scale, with 0–2 meaning “none“, “mild to moderate”, and “severe” symptom. A HAMD score of 8 to 20 may indicate depression; a score of 20 to 35 confirms depression; a score above 35 points indicates severe depression. HAMD reduction after antidepressant treatment indicates drug efficacy. The HAMD reduction rate is defined as (baseline score - score after treatment) / baseline score × 100% and is usually used to grade drug efficacy as remarkably effective (HAMD reduction rate ≥50%), effective (HAMD reduction rate ≥25%), or ineffective (HAMD reduction rate <25%). Depressed patients were assessed with HAMD within three days of hospital admission, and the controls were assessed with HAMD after the physical examination. The evaluations were carried out via conversation and observation by two trained attending physicians and scored by two raters independently with a consistency test Kappa of 0.76–0.89. HAMD was also administered to depressed patients after 8 weeks of antidepressant treatment.

### Paroxetine treatment regimens

All depression patients also signed informed consent for treatment with paroxetine (Sino-US Tianjin SmithKline Pharmaceutical Co., Ltd., China). Paroxetine is a new, first-line clinical antidepressant, and its mechanism is to inhibit reuptake of presynaptic 5-HT, resulting in a significant antidepressant effect while being highly safe (21,22). The starting dose of paroxetine was 10 mg/day via oral administration after breakfast. Depending on the patient's condition and the extent of drug resistance, the dose was increased to 20 mg/day in 5 to 7 days and to 30 mg/day before the second weekend (10 to 14 days), with a total duration of 8 weeks. Patient compliance increased after 8 weeks of treatment.

### Blood sample collection and processing

For control samples, 4 mL of venous blood was collected using an anticoagulant-free disposable vacuum tube under fasting state at the time of physical examination. For depressed patients, 4 mL of venous blood was collected using an anticoagulant-free disposable vacuum tube under fasting state on the day of HAMD scale evaluation. Another 4 mL of venous blood was collected from depressed patients after 8 weeks of antidepressant treatment. Blood samples were placed at room temperature to coagulate. After blood coagulation, samples were centrifuged at 1610 *g* at room temperature for 10 min. The supernatant was transferred to a microcentrifuge tube and stored at -80°C until quantitative real time polymerase chain reaction (qPCR) analysis. Blood samples with hemolysis were resampled.

### qPCR assay

Serum total RNA was extracted using the frozen serum samples according to kit instructions (Qiagen, USA). Extracted RNA samples were assayed for the 260/280 absorbance value using a UV spectrophotometer, and the RNA concentration was calculated before the samples were stored at -80°C for later use. The reverse transcription of cDNA was conducted following the kit instructions (Qiagen). Using the gene sequence database GenBank and the miR database BASE, we designed miRNA-451a, miRNA-34a-5p, and miRNA-221-3p specific reverse transcription primers with a stem-loop structure using Primer 5.0 primer design software (Table 1). Primers were synthesized by Shanghai Sangon Biotech Inc. (China). The qRT-PCR reaction system for miRNA-451a, miRNA-34a-5p, and miRNA-221-3p was 20 µL, including 10 µL of SYBR PremixExTaq, 0.4 µL of the Forward Primer, 0.4 µL of the Reverse Primer, 0.4 µL of ROX Reference Dye II, 2 µL of the DNA template, and 6.8 µL of dH_2_O. The reaction conditions were set to 95°C for 30 s, 95°C for 5 s, and 60°C for 30 s, for a total of 40 cycles. Using U6 as the internal control, reliability of the results was assessed using the PCR melting curve. The CT value (amplification power curve inflection point) was used to calculate the relative expression of target genes using 2^-△△^Ct (12).

**Table 1.** Primer sequences of miRNA-451a, miRNA-34a-5p, miRNA-221-3p, and U6 used in quantitative real time polymerase chain reaction (qPCR).

Gene	Forward	Reverse
U6	5′-CTCGCTTCGGCAGCACA-3′	5′-AACGCTTCACGAATTTGCGT-3′
miRNA-451a	5′-ACACTCCAGCTGGGAAACCGTTACCATTACT-3′	5′-CTGGTGTCGTGGAGTCGGCAA-3′
miRNA-34a-5p	5′-UGGCAGUGUCUUAGCUGGUUGU-3′	5′-AACCAGCUAAGACACUGCAAUU-3′
miRNA-221-3p	5′-AGCTAAAAAAGCTACATTGTCTGCTGGGTTTCG-3′	5′-GATCCGAAACCCAGCAGACAATGTAGCTTTTTT-3′

### Statistical analysis

We used SPSS18.0 statistical software (IBM; USA) for data analysis. Data are reported as means±SD. One-way analysis of variance (ANOVA) was used to compare means among multiple groups, and *t*-test was used for comparison between two groups. Correlation among variables was analyzed using the Pearson correlation test. P<0.05 was considered statistically significant.

## Results

### Characteristics of patients and controls

Seventy-eight controls were compared with 84 depressed patients. There were no significant differences between depressed patients and controls with regard to sex, age, education, or family history of depression (all P>0.05) (Table 2).

**Table 2.** Characteristics of cases and controls.

Variable	Controls	Cases	P
Number	78	84	
Gender			
Male	37	30	0.152
Female	41	54	
Age (Years)	35.51±13.62	39.21±13.07	0.080
Education^a^	6/25/20/27	7/27/21/29	0.999
Family history			
No	62	62	0.394
Yes	16	22	

Data are reported as means±SD or absolute numbers. ^a^Education was categorized into elementary, middle, high school, and college. Statistical analysis was done with the *t*-test or chi-squared test.

### Levels of miRNA-451a, miRNA-34a-5p, and miRNA-221-3p, and HAMD scores

**Figure 1.**
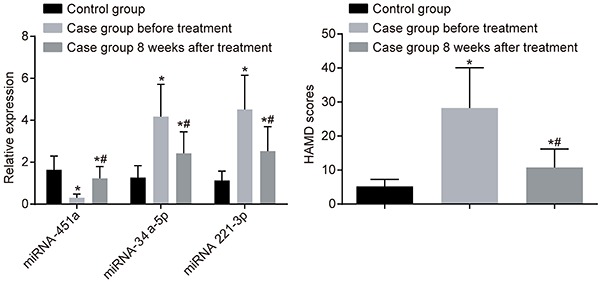
Left panel, eight weeks after paroxetine treatment, miRNA-451a expression was increased while miRNA-34a-5p and miRNA-221-3p expressions were decreased. Right panel, eight weeks after paroxetine treatment, the Hamilton Depression Scale (HAMD) scores were decreased. Data are reported as means±SD. *P<0.05 *vs* control group; ^#^P<0.05 *vs* case group before treatment (ANOVA).

qRT-PCR results showed that miRNA-451a expression levels were significantly decreased, and miRNA-34a-5p and miRNA-221-3p expression levels were significantly increased in depressed patients pre- and post-antidepressant treatment (all P<0.05) compared with the controls. For depressed patients, miRNA-451a levels increased significantly, while miRNA-34a-5p and miRNA-221-3p levels decreased significantly after 8 weeks of treatment (all P<0.05) (Figure 1A).

HAMD evaluation showed that depressed patients had significantly higher HAMD scores than controls (all P*<*0.05). HAMD scores significantly decreased in patients after 8 weeks of treatment (all P<0.05), as shown in Figure 1B.

### Correlation between HAMD scores and miRNA levels

**Figure 2.**
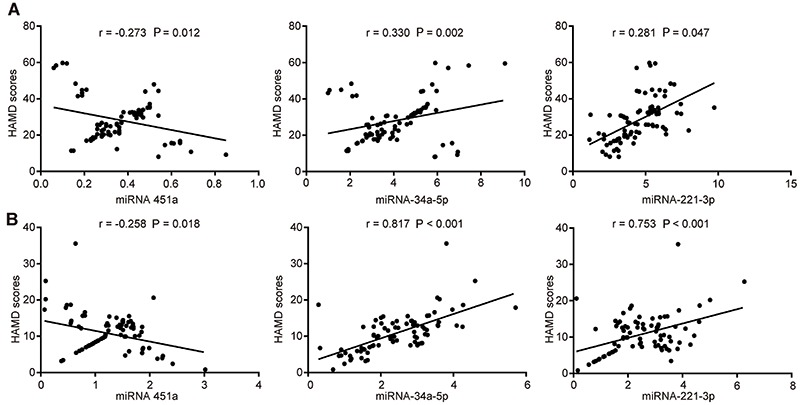
Pearson correlation analysis indicated that miR-451a expression was negatively correlated with the Hamilton Depression Scale (HAMD) scores, while miR-34a-5p and miR-221-3p expressions were positively correlated with HAMD scores before and after paroxetine treatment. Panel *A*, before paroxetine treatment. Panel *B*, after paroxetine treatment. r<0 indicates negative correlation while r>0 indicates positive correlation.

Pearson correlation tests of each miRNA and HAMD score showed that, before and after antidepressant treatment, miRNA-451a level was negatively correlated with HAMD score (r*<*0, P<0.05), while miRNA-34a-5p and miRNA-221-3p levels were positively correlated with HAMD scores (all r>0, all P<0.05), as shown in Figure 2.

### miRNA levels according to paroxetine responses

Based on the HAMD reduction rate, the antidepressant therapy was deemed remarkably effective (HAMD reduction rate ≥50%) for 50 cases, effective (HAMD reduction rate ≥25%) for 25 cases, and ineffective (HAMD reduction rate <25%) for 9 cases. Analysis of miRNA expression levels in different efficacy groups showed no significant difference before treatment (all P>0.05). However, miRNA-451a was significantly elevated (all P<0.05), while miRNA-34a-5p and miRNA-221-3p levels declined significantly in the markedly effective and moderately effective groups (all P<0.05). There were no significant changes in miRNA-451a, miRNA-34a-5p, and miRNA-221-3p in the ineffective group (all P>0.05). Compared with the markedly effective group, post-treatment miRNA-451a levels were significantly lower (P<0.05), and the miRNA-34a-5p and miRNA-221-3p levels were higher in the moderately effective and ineffective groups (all P<0.05). Compared with the moderately effective group, post-treatment miRNA-451a levels were significantly lower (P<0.05), and the miRNA-34a-5p and miRNA-221-3p levels were significantly higher in the ineffective group (all P<0.05), as shown in Table 3.

**Table 3.** Relative serum miRNA-451a, miRNA-34a-5p, and miRNA-221-3p levels differed when patients showed different response to antidepressants.

miRNA	Before treatment	After treatment
Markedly effective group (n = 50, HAMD reduction rate ≥50%)		
miRNA-451a	0.34±0.15	1.40±0.50^+^
miRNA-34a-5p	3.89±1.19	2.00±0.73^+^
miRNA-221-3p	4.80±1.50	2.06±0.83^+^
Effective group (n = 25, HAMD reduction rate ≥25%)		
miRNA-451a	0.27±0.18	1.14±0.57^+^*
miRNA-34a-5p	4.55±1.48	2.67±0.99^+^*
miRNA-221-3p	4.25±1.73	2.81±1.12^+^*
Ineffective group (n = 9, HAMD reduction rate <25%)		
miRNA-451a	0.29±0.18	0.53±0.29*^#^
miRNA-34a-5p	4.74±2.85	4.05±0.72*^#^
miRNA-221-3p	3.73±1.84	4.38±0.82*^#^

Data are reported as means±SD. ^+^P<0.05 compared with pre-treatment; *P<0.05 compared with the markedly effective group after treatment; ^#^P<0.05 compared with the effective group after treatment (ANOVA). miRNA: microRNA; HAMD: 24-item Hamilton Depression Scale.

### Association of miRNA levels with the course of disease and suicide attempts

Serum miRNA-451a, miRNA-34a-5p, and miRNA-221-3p were not associated with sex, family history, suicidal ideation, first and recurrent depression, or early-onset and late-onset depression before antidepressant treatment (all P>0.05), but were significantly associated with the disease course and history of suicide attempts (all P<0.05), as shown in Table 4. After treatment, there was no significant association of serum miRNA-451a, miRNA-34a-5p or miRNA-221-3p with sex, family history, suicidal ideation, first and recurrent depression, early- and late-onset depression, disease course, or history of suicide attempts (all P>0.05), as shown in Table 5.

**Table 4.** Relationships between relative serum miRNA-451a, miRNA-34a-5p, and miRNA-221-3p levels and suicidal behavior before treatment.

	miRNA-451a	P	miRNA-34a-5p	P	miRNA-221-3p	P
Gender		0.230		0.173		0.471
Male	0.28±0.18		3.87±1.79		4.69±2.05	
Female	0.33±0.17		4.35±1.37		4.42±1.36	
Family history		0.674		0.159		0.660
Yes	0.30±0.20		3.78±1.72		4.39±1.91	
No	0.32±0.16		4.32±1.46		4.57±1.54	
Suicide attempts		0.014		<0.001		0.0110
Yes	0.23±0.16		5.30±1.78		5.35±1.71	
No	0.34±0.17		3.85±1.31		4.28±1.54	
Suicidal ideation		0.217		0.358		0.599
Yes	0.28±0.16		3.96±1.80		4.39±1.92	
No	0.33±0.18		4.29±1.40		4.59±1.48	
Course of disease		0.003		0.012		<0.001
≥5 years	0.26±0.16		4.56±1.70		5.10±1.67	
<5 years	0.37±0.17		3.72±1.18		3.82±1.27	
Type of disease						
First	0.34±0.17	0.111	4.28±1.26	0.517	4.26±1.33	0.110
Recurrent	0.28±0.17		4.06±1.82		4.83±1.89	
Disease onset						
Late-onset (≥30 years)	0.30±0.18	0.626	4.05±1.78	0.604	4.97±2.18	0.091
Early-onset (<30 years)	0.32±0.1 7		4.24±1.43		4.32±1.28	

Data are reported as means±SD (*t*-test). miRNA, microRNA.

**Table 5.** Relationships between relative serum miRNA-451a, miRNA-34a-5p, and miRNA-221-3p levels and suicidal behavior 8 weeks after treatment.

	miRNA-451a	P	miRNA-34a-5p	P	miRNA-221-3p	P
Gender		0.355		0.612		0.603
Male	1.15±0.67		2.34±1.19		2.44±1.35	
Female	1.27±0.50		2.46±0.94		2.58±1.07	
Family history		0.832		0.587		0.608
Yes	1.25±0.59		2.32±1.20		2.42±1.36	
No	1.22±0.56		2.46±0.97		2.57±1.10	
Suicide attempts		0.068		0.46		0.455
Yes	1.44±0.80		2.57±1.45		2.71±1.65	
No	1.17±0.47		2.37±0.88		2.48±1.00	
Suicidal ideation		0.706		0.077		0.077
Yes	1.19±0.63		2.14±1.20		2.21±1.37	
No	1.24±0.54		2.56±0.91		2.6 9±1.04	
Course of disease		0.073		0.084		0.087
≥5 years	1.33±0.63		2.60±1.15		2.73±1.31	
<5 years	1.11±0.44		2.21±0.83		2.29±0.94	
Type of disease						
First	1.14±0.44	0.124	2.57±0.81	0.143	2.70±0.92	0.149
Recurrent	1.33±0.67		2.24±1.22		2.33±1.39	
Disease onset						
Late-onset (≥30 years)	1.30±0.65	0.41	2.48±1.16	0.713	2.59±1.32	0.747
Early-onset (<30 years)	1.19±0.52		2.39±0.97		2.50±1.11	

Data are reported as means±SD (*t*-test). miRNA, microRNA.

### Association of HAMD scores with disease course and suicide attempts

Pre-treatment HAMD score was not associated with sex, family history, suicidal ideation, first and recurrent depression, or late-onset and early-onset depression (all P>0.05), but was significantly associated with disease course and history of suicide attempts (all P<0.05). After 8 weeks of treatment, HAMD scores were not associated with sex, family history, suicidal ideation, first and recurrent depression, late- and early-onset depression, course of disease, or history of suicide attempts (all P>0.05), as shown in Table 6.

**Table 6.** Relationships between HAMD scores and suicidal behavior of patients with depression before and 8 weeks after treatment.

	Before treatment	P	8 weeks after treatment	P
Gender		0.372		0.289
Male	29.77±14.09		9.92±5.40	
Female	27.34±10.49		11.23±5.39	
Family history		0.486		0.374
Yes	26.68±14.11		11.65±7.59	
No	28.75±11.05		10.45±4.42	
Suicide attempts		0.044		0.238
Yes	32.93±14.90		12.05±6.44	
No	26.82±10.58		10.39±5.05	
Suicidal ideation		0.416		0.217
Yes	26.71±13.43		9.73±5.63	
No	28.96±11.07		11.28±5.26	
Course of disease		0.005		0.155
≥5 years	31.46±11.99		11.53±5.16	
<5 years	24.26±10.60		9.84±5.61	
Type of disease				
First	27.90±11.12	0.801	11.43±5.15	0.288
Recurrent	28.56±12.83		10.00±5.64	
Disease onset				
Late-onset (≥30 years)	31.02±13.30	0.148	10.54±5.28	0.798
Early-onset (<30 years)	26.95±11.07		10.87±5.50	

Data are reported as means±SD (*t*-test). HAMD, 24-item Hamilton Depression Scale.

## Discussion

This study aimed to explore the associations of miRNA-451a, miRNA-34a-5p, and miRNA-221-3p with antidepressant drug efficacy. Our findings suggest that patients with depression had decreased serum miRNA-451a levels and increased miRNA-34a-5p and miRNA-221-3p levels, which are closely related to the therapeutic efficacy of antidepressant drugs. These miRNAs have the potential to become biomarkers for early diagnosis and therapeutic efficacy for depression.

The results of this study showed that, compared with controls, depressed patients had reduced expression of miRNA-451a but increased miRNA-34a-5p and miRNA-221-3p expression levels, which can be reversed with antidepressant treatment. It has been shown that a large number of miRNA are specifically expressed or enriched in the brain or central nervous system, and as a neurological disorder, depression may lead to disordered miRNA expression (23). In addition, the turnover of miRNA in neurons is faster than in other cell types, suggesting that the neuronal miRNA system could result in the rapid adaptation to neuronal activity and be associated with the calpain-dependent activation (24). Our findings are consistent with those of Wan et al. (25), who also observed reduced expression of miRNA-451a, and increased expression of miRNA-221-3p in depression patients. Additionally, miRNA-451a was demonstrated to work as a candidate biomarker for depression based on the mechanism of action of ketamine (26). It was reported that overexpression of miRNA-34a can lower brain-derived neurotrophic factor (BDNF) expression (27), which is considered one of the major etiologic mechanisms of depression (28). In addition, high expression of miRNA-34a can reduce the expression of the SIRT1 gene (29), which may also contribute to the pathogenesis of depression. Thus, changes in miRNA expression could affect the expression of many genes related to neural activity in the brain, leading to the onset and development of depression.

Our results also suggested that 8 weeks of antidepressant treatment could significantly increase miRNA-451a expression, decrease miRNA-34a-5p and miRNA-221-3p expression, and decrease HAMD scores. Previous studies showed that antidepressants can lower miRNA-221-3p (16) and miRNA-34a-5p (17) levels, which is consistent with the results of our study. Furthermore, comparison among groups with different levels of treatment efficacy showed that the increase in miRNA-451a levels and the decrease in miRNA-34a-5p and miRNA-221-3p levels are positively associated with higher antidepressant efficacy. It was reported that miRNA-221 in serum of patients with major depressive disorder was down-regulated after treatment, indicating that antidepressant treatment has a normalizing effect on the circulating miRNA levels (30). Moreover, miRNA-34c-5p has been previously demonstrated to affect the basic mechanisms of brain neuroplasticity and stress response (31). A study of the relationship between early treatment outcome and suicidal ideation in 705 cases of hospitalized depression patients using HAMD score showed that the incidence of suicide ideation was 3-5 times higher in patients who had low treatment efficacy than in patients who had high treatment efficacy and that early treatment efficacy significantly reduced pessimism (32). Therefore, it is critical to evaluate treatment efficacy in a timely manner. Based on the above findings, miRNA-451a, miRNA-34a-5p, and miRNA-221-3p could become potential markers for early diagnosis and therapeutic efficacy.

The results also showed that serum miRNA-451a, miRNA-34a-5p, and miRNA-221-3p expression was closely associated with the disease course and suicide attempts. Patients with ≥5 years of depression have different miRNA-451a, miRNA-34a-5p, and miRNA-221-3p expression than those diagnosed for fewer than 5 years. A previous study has shown that a longer course of depression is associated with worse cognitive dysfunction, and loss of interest, self-efficacy, and awareness (33). Since miRNA may regulate central nervous system functions such as cognitive function and reward feedback, we hypothesized that with the extension of disease course, changes in miRNA-451a, miRNA-34a-5p, and miRNA-221-3p expression may cause the deterioration of the central nervous system. Studies have shown that reduced BDNF plays an important role in depression and suicidal behavior (34). Kim et al. (35) and Lee et al. (36) found that patients with suicidal behavior have lower plasma BDNF than those without suicidal behavior. As previously mentioned, changes in miRNA expression affects the expression of a number of genes related to neural activity in the brain, and so we hypothesize that the low expression of BDNF in patients with a history of suicide attempts is caused by disordered expression of miRNA-451a, miRNA-34a-5p, and miRNA-221-3p. Our study found no significant association of serum expression of these miRNAs with suicidal ideation, but there was a significant association with suicide attempts, suggesting the abnormal expression of serum miRNA is more likely to contribute to suicide behavior rather than suicide ideation.

There are several limitations in our study. First, the sample size was relatively small; second, some clinical characteristics and medical history that may influence the outcome were not well matched among all enrolled depression patients; third, the miRNA quantification platforms and sample type need further standardization; fourth, although paroxetine is a new, first-line clinical antidepressant with considerable antidepressant effect and high safety, other antidepressants should be assessed on the same correlations with serum miRNA-451a, miRNA-34a-5p, and miRNA-221-3p levels. Due to these restrictions, the use of serum circulating miRNA-451a, miRNA-34a-5p, and miRNA-221-3p as predictors for assessing antidepressant treatment requires further investigation.

In conclusion, depression patients have reduced serum miRNA-451a levels but increased miRNA-34a-5p and miRNA-221-3p expression levels. The expression levels of these miRNAs are closely related to the efficacy of antidepressant drugs.
